# Engineering nanoparticles-enabled tumor-associated macrophages repolarization and phagocytosis restoration for enhanced cancer immunotherapy

**DOI:** 10.1186/s12951-024-02622-1

**Published:** 2024-06-18

**Authors:** Yonghua Gong, Wenyue Gao, Jinyang Zhang, Xia Dong, Dunwan Zhu, Guilei Ma

**Affiliations:** Key Laboratory of Biomaterials and Nanotechnology for Cancer Immunotherapy, The Tianjin Key Laboratory of Biomaterials, Institute of Biomedical Engineering, Peking Union Medical College & Chinese Academy of Medical Sciences, Tianjin, 300192 China

**Keywords:** Nanoparticles, Tumor-associated macrophages, Combination, Phagocytosis, Polarization, Cancer immunotherapy

## Abstract

Tumor-associated macrophages (TAMs) are pivotal within the immunosuppressive tumor microenvironment (TME), and recently, have attracted intensive attention for cancer treatment. However, concurrently to promote TAMs repolarization and phagocytosis of cancer cells remains challenging. Here, a TAMs-targeted albumin nanoparticles-based delivery system (M@SINPs) was constructed for the co-delivery of photosensitizer IR820 and SHP2 inhibitor SHP099 to potentiate macrophage-mediated cancer immunotherapy. M@SINPs under laser irradiation can generate the intracellular reactive oxygen species (ROS) and facilitate M2-TAMs to an M1 phenotype. Meanwhile, inhibition of SHP2 could block the CD47-SIRPa pathway to restore M1 macrophage phagocytic activity. M@SINPs-mediated TAMs remodeling resulted in the immunostimulatory TME by repolarizing TAMs to an M1 phenotype, restoring its phagocytic function and facilitating intratumoral CTLs infiltration, which significantly inhibited tumor growth. Furthermore, M@SINPs in combination with anti-PD−1 antibody could also improve the treatment outcomes of PD−1 blockade and exert the synergistic anticancer effects. Thus, the macrophage repolarization/phagocytosis restoration combination through M@SINPs holds promise as a strategy to concurrently remodel TAMs in TME for improving the antitumor efficiency of immune checkpoint block and conventional therapy.

**Supplementary Information:**

The online version contains supplementary material available at 10.1186/s12951-024-02622-1.

## Introduction

Tumor-associated macrophages (TAMs) in the tumor microenvironment (TME) are the most abundant immune cells, playing a critical role in the prognosis of tumor [1, 2]. In the TME, activated macrophages usually differentiate into two categories of antitumoral M1 phenotype and protumoral M2 phenotype [3]. TAMs within TME are mainly the M2 phenotype, which secrete immunosuppressive cytokines and attenuate phagocytosis activity to subvert the antitumor immunity [4, 5]. TAMs are greatly associated with immunosuppression, tumor metastasis and therapeutic resistance in most solid tumor types. Considering the plasticity of TAMs, therapeutic strategies that can restore antitumor immunity of macrophages in TME may offer great therapeutic potential for cancer [6–8]. 

To date, various nanomedicine-based strategies for promoting the polarization of M2-TAMs to M1 phenotype to boost the antitumor immunity have been developed. For example, toll-like receptors agonists, transcription signal modulators, plasmid DNA, or chemical compounds have been loaded into nanoparticles to enhance antitumor therapy by reprogramming M2-TAMs into functional M1-like macrophages [9–12]. Moreover, in several recent studies suggested that intracellular reactive oxygen species (ROS) generation can re-educate the M2-TAMs to M1 phenotype by activating ROS/NF-kB signal pathway [13, 14]. Chen and colleagues shifted TAMs from the M2 to the M1 phenotype using nanoparticles-based ROS photogeneration with high conversion efficiency [15]. Liang et al. developed MnO_2_ nanosheets to produce plenty of the intracellular ROS for altering macrophage polarization and improving TAMs-based antitumor immunity [13]. However, although reprogramming TAMs polarization is essential for macrophage-based cancer immunotherapy, macrophages are also important immune cells with the role in phagocytizing, and the immunotherapeutic effect was still confined by the poor phagocytic behavior of macrophages [16–18]. 

Tumor cells may express high levels of CD47 on their surface, which can bind with signal regulatory protein alpha (SIRPa) receptor on macrophages, resulting in a “don’t eat me” signal and phagocytosis inhibition [19, 20]. CD47-SIRPa axis-blocking agents such as Hu5F9-G4, ALX148, and SHP099 (a SHP-2 inhibitor) have been widely applied for cancer treatment [21–24]. Although promising, given that the broad expression of CD47 in healthy cells, systemic infusion of CD47 inhibitors can cause the limited efficacy and adverse effects such as anemia [25, 26]. To tackle these limitations, some nanodrug delivery systems are designed to target TAMs in the tumor tissues [27, 28]. Zang et al. developed a CD206 (macrophage mannose receptor 1) targeting nanoparticle modified with mannose, which has TAMs-targeted capacity, effectively depleted TAMs and eventually restrained tumor growth without eliciting systemic effects [29]. Additionally, nanoparticles as co-delivery systems can realize the synchronized delivery of two drugs into the same cell over the randomized drug distribution of two drugs [30–32]. In this case, developing TAMs-tageted nanomedicines that can simultaneously reprogram M2-TAMs into functional M1 phenotype and restore phagocytosis would be a promising strategy to concurrently remodel TAMs for enhancing cancer immunotherapy efficacy.

**Scheme 1:**
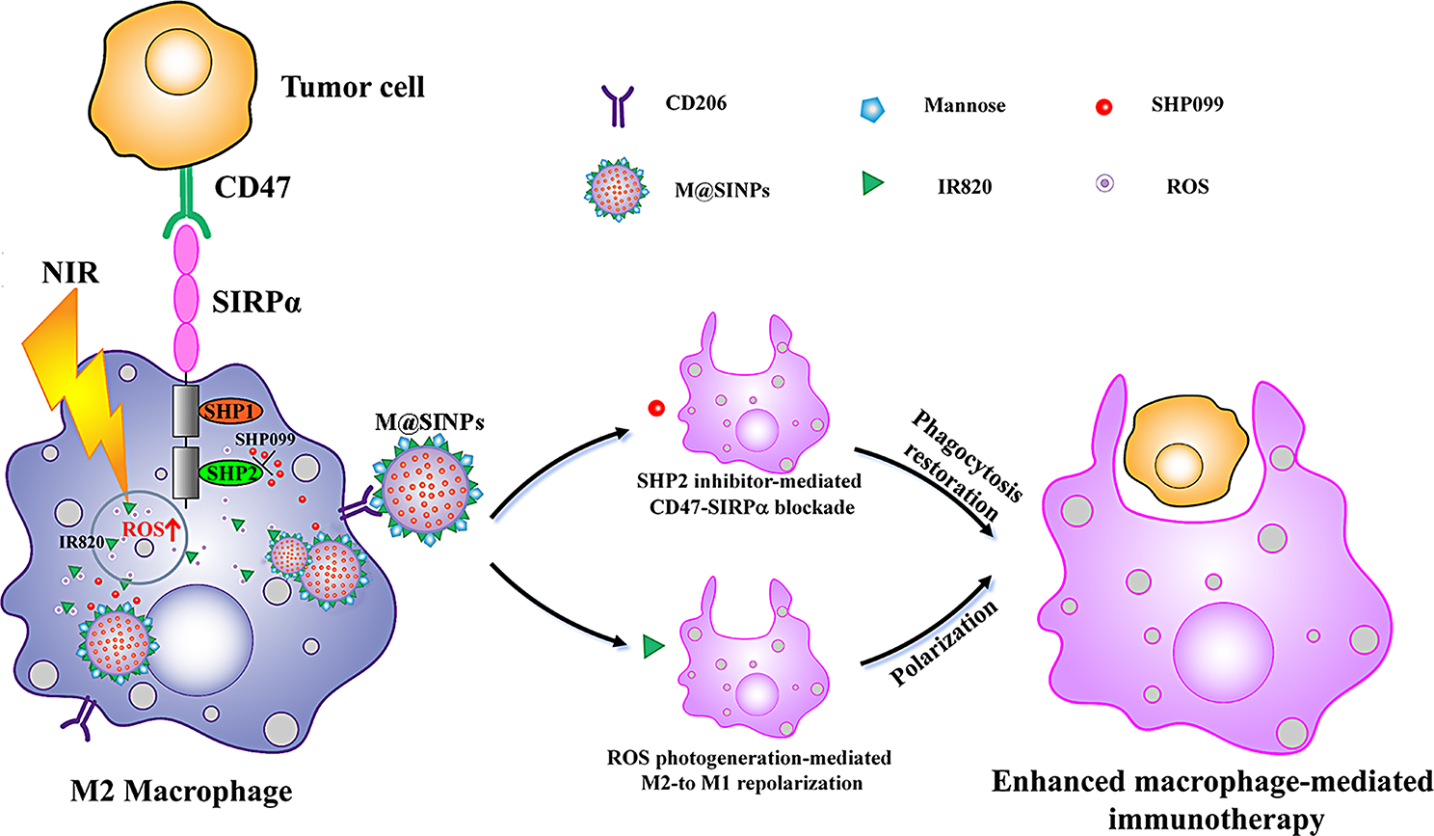
Schematic illustration of M@SINPs-mediated M2-to-M1 repolarization and phagocytosis restoration of TAMs for improved cancer immunotherapy

Herein, we engineer a mannose-modified albumin-based nanoparticle (M@SINPs) to co-deliver dual modulators for targeting two distinct mechanisms involved TAMs activation, namely using the ROS photogenerator IR820 to stimulate M2-to-M1 repolarization of TAMs and the small molecule SHP-2 inhibitor SHP099 to block CD47-SIRPa axis to promote macrophage phagocytosis (Scheme 1). Both IR820 and SHP099 are loaded into one nanoparticle for TAMs-targeted delivery to the same cell will favour simultaneously reprogramming M2-TAMs into M1 phenotype and restoring its phagocytic function. Additionally, previous findings suggested that the limited PD-1/PD-L1 blockade therapy is due to the “T cell exhaustion” state in the immunosupressive TME that resulting in the progressive tumor growth [33, 34]. Studies have shown that TAMs as immunosuppressive cells are highly implicated in the suppression of antitumor T cell function [35, 36]. In this study, we investigate whether M@SINPs could be used as an immune regulator to improve macrophage-mediated cancer immunotherapy. We also discuss whether the M@SINPs-mediated TAMs remodulation could be critical for potentiating the antitumor immunotherapy efficacy of anti-PD-1 antibody (aPD-1).

## Results and discussion

### Preparation and characterization of M@SINPs

**Fig. 1:**
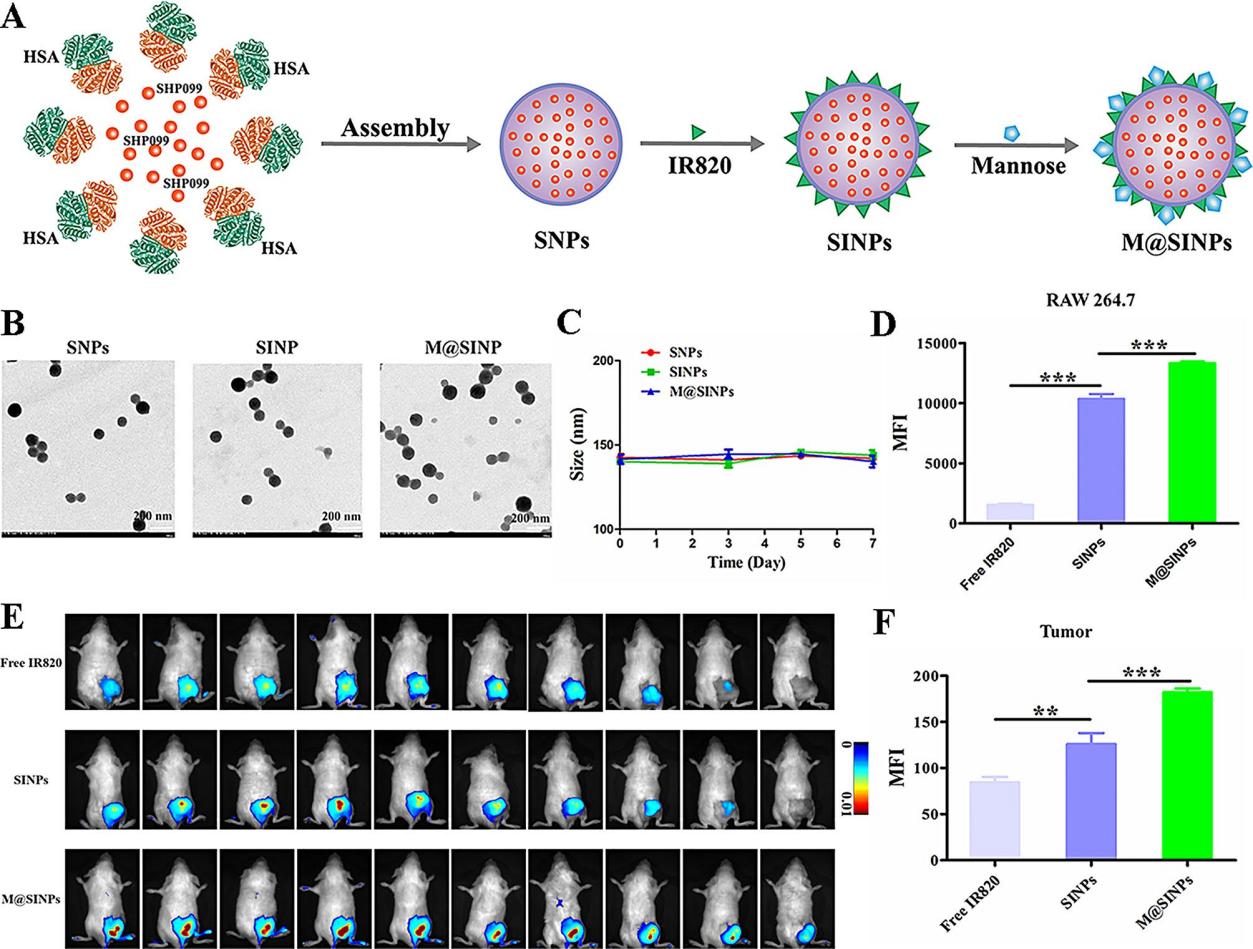
(**A**) Construction of M@SINPs. (**B**) TEM images of SNPs, SINPs and M@SINPs. (**C**) Stability assay of SNPs, SINPs and M@SINPs in 10% FBS over time (*n* = 3). (**D**) FCM analysis of the cellular internalization of free IR820, SINPs and M@SINPs in RAW264.7 cells (*n* = 3). (**E**) In vivo imaging of free IR820, SINPs and M@SINPs after *i.v.* injection at indicated time points. (**F**) Ex vivo fluorescence analysis in the tumors tissues collected at 12 h post-treatment (*n* = 3). (**p < 0.01, ***p < 0.001)

The preparation of M@SINPs briefly contained two steps as illustrated in Fig. 1A. HSA and SHP099 were firstly assembled into SHP099-loaded HSA nanoparticles (SNPs) using a desolvation method [37]. After that, the obtained SNPs were sequentially IR820 loaded (SINPs) and mannose modified to prepare the final M@SINPs. Representative transmission electron microscope (TEM) images showed the spherical morphology of the prepared nanoparticles with a uniform distribution (Fig. 1B). Dynamic light scattering determination revealed that SNPs, SINPs and M@SINPs all had an average diameter around 140 nm with a polydispersity index (PDI) of ~ 0.1 (Table S1). Meanwhile, the zeta potential of SINPs changed after mannose modification, which further indicated the successful conjugation of mannose (Figure S1). The loading of SHP099 and IR820 for M@SINPs was 43.2 ± 0.3 and 24.7 ± 0.1 µg mg^− 1^ with the loading efficiency of 86.4% and 98.9%, respectively, by high-performance liquid chromatography (HPLC) and UV detection (Table S1). The obtained M@SINPs remained stable in the size for 7 days upon incubation with 10% fetal bovine serum (FBS) (Fig. 1C). Then, we analyzed the drug release kinetics profiles of these nanoparticles, and all nanoparticles displayed a sustained release of SHP099 and IR820, respectively (Figure S2).

Next, we evaluated the ability of mannose modification to increase the intracellular internalization of M@SINPs in M2 polarized RAW264.7 macrophages (M2-RAW264.7). For the study of the intracellular uptake of the nanoparticles by macrophages, free IR820, SINPs and M@SINPs were incubated with M2-RAW264.7 for 4 h, and the intracellular internalization of the nanoparticles was evaluated quantitatively and qualitatively. Figure 1D showed that M@SINPs were more efficiently internalized by M2-RAW264.7 (about 1.3 times as much as the SINPs). Confocal laser scanning microscopy (CLSM) observation further confirmed this result. As shown in Figure S3, compared with free IR820 and SINPs, the cells incubated with M@SINPs showed more obvious fluorescence signals.

Furthermore, we studied the in vivo tumor accumulation of M@SINPs in CT26-bearing mice. As compared with free IR820 and SINPs, M@SINPs exhibited a prolonged tumor retention time and the stronger fluorescence signal of M@SINPs still could be observed even at 120 h after injection (Fig. 1E). The fluorescence intensity of IR820 at tumor site was 2.20- and 1.44-fold higher in the M@SINPs group than free IR820 and SINPs at 12 h after the injection (Fig. 1F), respectively, suggesting that M@SINPs has relatively better tumor targeting properties. In addition, tumor tissues were also harvested for assessment whether TAMs can be targeted by *i.v.* M@SINPs administration using flow cytometry (FCM). Figure S4 shows that the M@SINPs can enhance intracelluar internalization of the nanoaprticles in TAMs in tumor site. Overall, the results demonstrated relatively good tumor-targeting properties of M@SINPs in vitro and in vivo, respectively.

### M@SINPs facilitated macrophages phagocytosis and M2-to-M1 repolarization

**Fig. 2:**
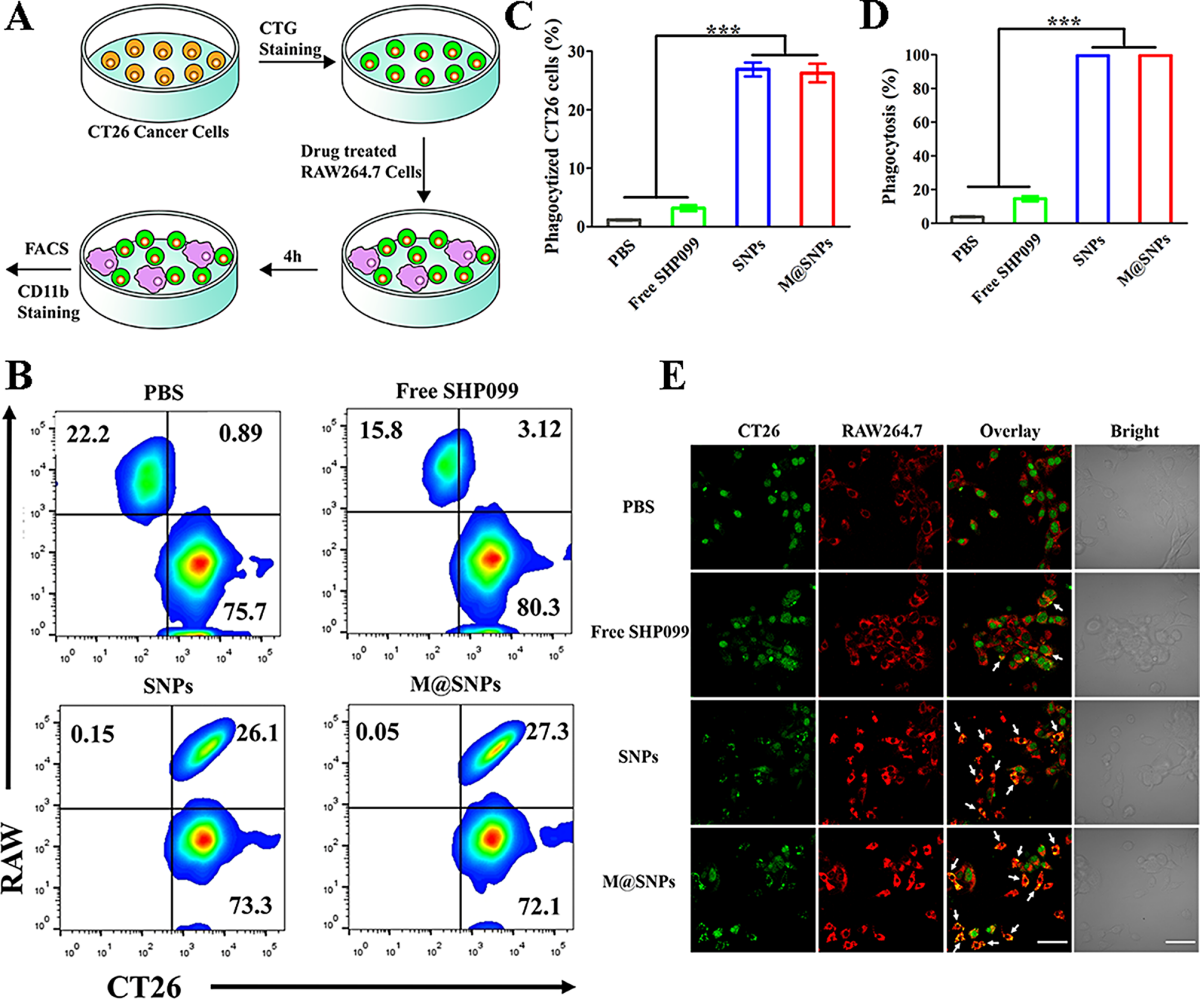
M@SINPs for increasing macrophages phagocytosis. (**A**) Schematic description of cell phagocytosis experiment. (**B**-**D**) FCM analysis of the phagocytosis of CT26 cells by RAW264.7 after treatment with free SHP099, SNPs or M@SINPs (*n* = 3). (**E**) Fluorescence images showing the phagocytosis of CTG-labeled CT26 cells (green) by Rhodaming B-labeled RAW264.7 (red). Scale bar, 50 μm. (***p < 0.001)

SHP099, as a SHP2 allosteric inhibitor, has shown promising results in facilitating the engulfment of tumor cells by macrophages in the previous works [23, 24]. To determine whether SHP099-loaded HSA nanoparticles could promote the phagocytosis by macrophages, CT26 cells were marked with cell tracker green (CTG, green) and then co-cultured with RAW264.7 in vitro, which had been pre-treated with the different SHP099 formulations (Fig. 2A). As detected by FCM analysis, compared with the control and free SHP099 group, more cancer cells were phagocytosed by RAW264.7 macrophages pre-incubated with SNPs and M@SNPs (Fig. 2B-D), thus indicating that SHP099-loaded HSA nanoaprticles can promote the phagocytosis of tumor cells by macrophages. Furthermore, confocal imaging further revealed that SNPs and M@SNPs significantly facilitated the phagocytosis of CT26 cells by RAW264.7 macrophages (Fig. 2E).

**Fig. 3:**
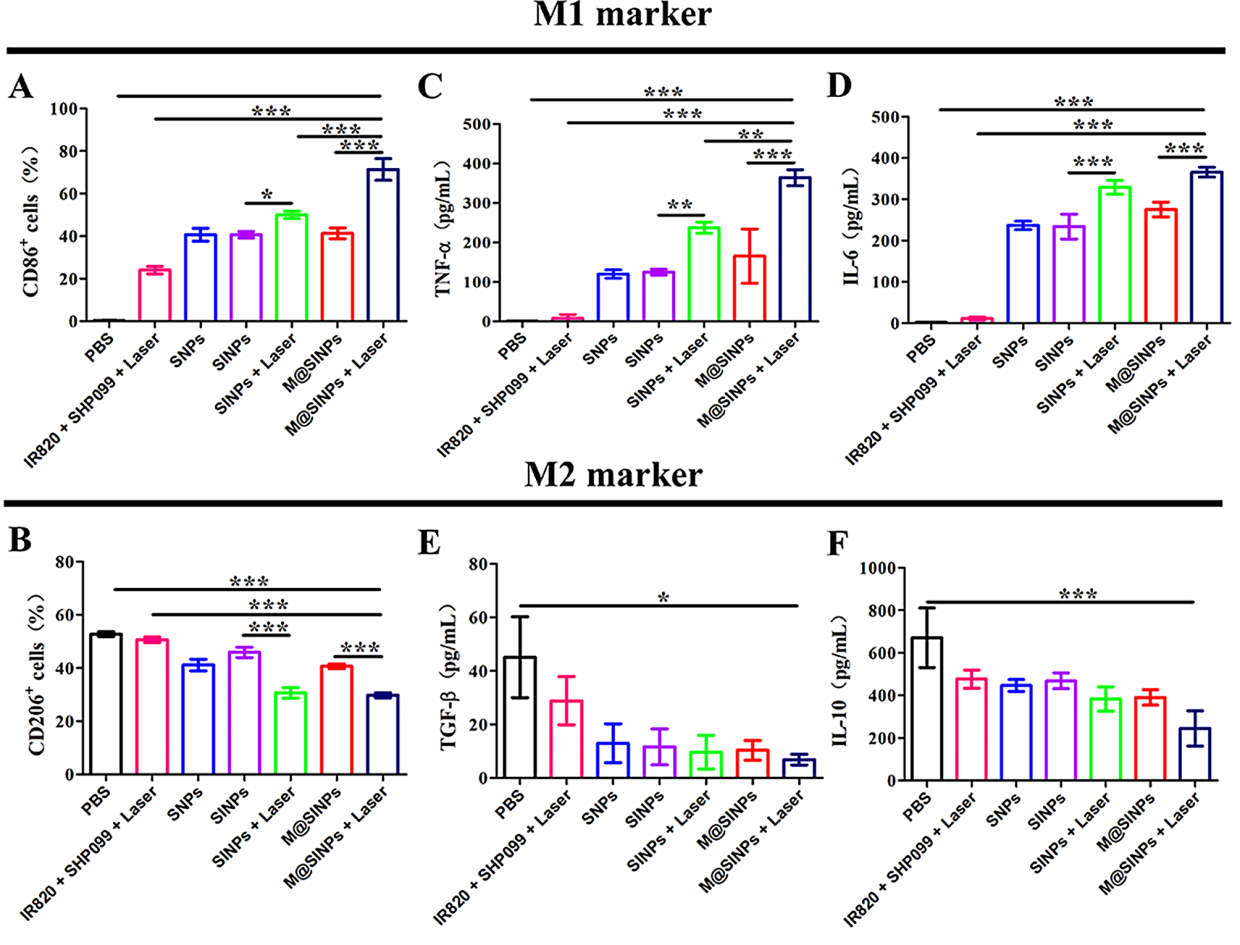
M@SINPs for reprogramming M2-to-M1 repolarization in vitro. (**A** to **B**) FCM analysis of the expression of M1 markers (CD86^+^) and M2 markers (CD206^+^) in M2-RAW264.7 with the treatments as indicated (*n* = 3). (**C**-**F**) Cytokines levels of TNF-α, IL-6, TGF-β and IL-10 in the culture medium of M2-RAW264.7 with the treatments as indicated (*n* = 3). (*p < 0.05, **p < 0.01, ***p < 0.001)

Intracellular ROS generation can affect the macrophage polarization and activation *via* the ROS/NF-kB signal pathway [13, 14]. To explore whether the prepared nanoparticles could repolarize M2-TAMs to M1 phenotype, we first evaluated the possible toxicity of the prepared nanoparticles and intracelluar ROS generation in RAW264.7 cells under laser irradiation. For cytotoxicity assay, RAW264.7 cells were incubated with free IR820, free SHP099, SNPs, SINPs and M@SINPs, and then irradiated for different time under laser irradiation (808 nm, 0.5 W/cm^2^). As indicated in Figure S5, all the formulations remained viable more than 80% under laser irradiation (0.5 W/cm^2^, 90s). Furthermore, we investigated the intracellular ROS generation in RAW264.7 cells using 2’,7’-dichlorodihydrofluorescein diacetate (DCFH-DA) probe. As shown in Figure S6, SNPs group showed no obvious green fluorescence. SINPs group and M@SINPs group showed dramatically increased green fluorescence relative to free IR820 group, thus confirming the effective intracelluar ROS photo-generation in RAW264.7 cells treated with the prepared IR820-loaded nanoparticles.

Upon confirmation of the intracelluar ROS photo-generation, we next evaluated the role played by the prepared nanoparticles on influencing macrophages polarity. Macrophages can express specific phenotypic biomarkers. For example, CD86, IL-6, iNOS and TNF-a were considered as M1-phenotypic biomarkers, whereas CD206, TGF-b, and IL-10 were considered as M2-phenotypic biomarkers. In this study, SINPs and M@SINPs treatments, upon 808 nm laser irradiation (0.5 W/cm^2^, 90 s), showed the increased expression levels of CD86, TNF-α and IL-6 and the reduced expression levels of CD206, TGF-β and IL-10 compared with SINPs and M@SINPs groups without laser irradiation, respectively (Fig. 3). These comparisons suggested that the intracellular ROS photogeneration could facilitate the M2-to-M1 repolarization of RAW264.7 cells. As expected, M@SINPs treatment could potentially repolarize RAW264.7 cells to M1 phenotype with the highest M1-biomarkers expression levels and the lowest M2-biomarker expression levels among all treatment groups.

### In vivo antitumor efficacy of M@SINPs

**Fig. 4:**
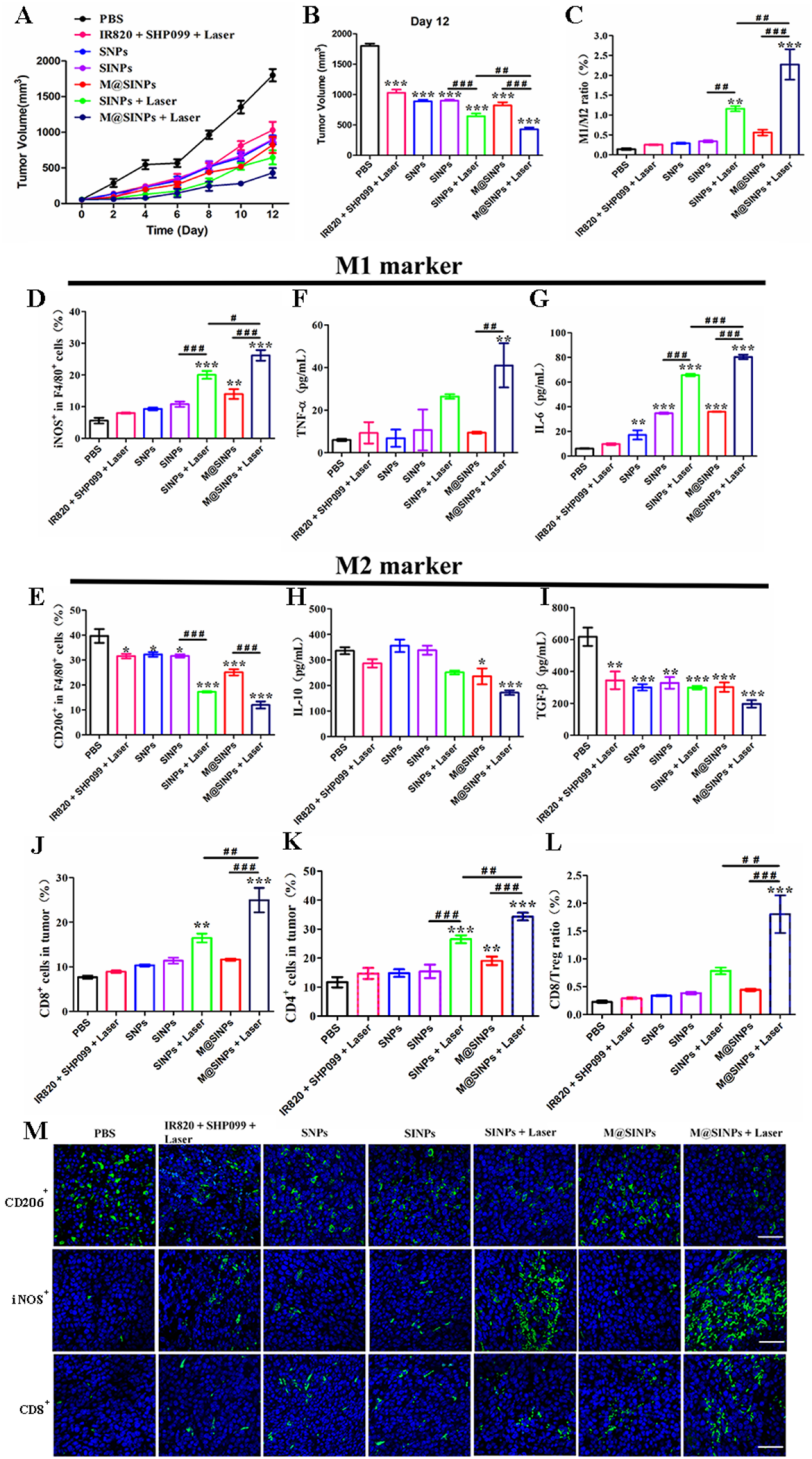
In vivo antitumor therapeutic evaluations of M@SINPs. (**A** and **B**) Tumor volume changes of mice after the indicated treatments (*n* = 5). (**C**-**E**) FCM analysis of the intratumoral M1 macrophage (iNOS^+^), M2 macrophage (CD206^+)^ and the ratio of M1/M2 (*n* = 3). (**F**-**I**) Cytokines levels in serum analyzed by ELISA kits (*n* = 3). (**J** and **K**) FCM analysis of the intratumoral CD8^+^ T cells and CD4^+^ T cells (*n* = 3). (**L**) The ratio of CD8/Treg in the tumor form the treated mice (*n* = 3). (**M**) Representative immunohistochemical images of tumor sections showing the infiltration of CD206^+^, iNOS^+^ and CD8 ^+^ cells (scale bar = 20 μm). DAPI was used to stain the nucleus of the cell (blue). (#< 0.05, ## < 0.01, ### < 0.001; * vs. PBS, *p < 0.05, **p < 0.01, ***p < 0.001)

Encouraged by the results from our in vitro studies, the antitumor ability of M@SINPs was further evaluated in CT26-bearing mice. The mice were *i.v.* injected with all formulations, and then under laser irradiation at 12 h post-injection as described in the Methods section. Compared to PBS group, SNPs, SINPs and M@SINPs group without laser irradiation groups induced a significant tumor growth inhibition (Fig. 4A and B). Previous reports have also demonstrated the effect of SHP099-mediated SHP-2 inhibition in controlling tumor growth in vitro and in vivo, which are consistent with our study [38–40]. More importantly, combination therapy with M@SINPs and laser irradiation (M@SINPs + Laser group) showed the greatest antitumor efficiency, attributed to the mannose-mediated targeted drug delivery and the therapeutic performance by simultaneous phagocytosis and polorization macrophages. Additionally, no apparent body weight loss and no obvious histopathological organ damage were observed in the M@SINPs-treated mice, suggesting no apparent toxicities caused by *i.v.* injected M@SINPs (Figure S7 and S8).

To validate the antitumor immune response induced by M@SINPs, immune cell populations at the tumor sites were firstly tested. M1 macrophages is crucial to stimulate the infiltration of intratumoral CD8^+^ cytotoxic T lymphocytes (CTLs) and hinder the immunosuppressive function of regulatory T cells (Tregs) [41]. Herein, the polarization effect of M@SIPNs were evaluated using FCM in the CT26-bearing mice after the various treatments. We found that intratumoral TAMs infiltration was significantly influenced by the SINPs or M@SINPs with laser treatment, with both exhibiting higher percentage of M1 macrophage (CD45^+^F4/80^+^iNOS^+^) and lower percentage of M2 mcarophage (CD45^+^F4/80^+^CD206^+^) when compared to SINPs or M@SINPs without laser irradiation (Fig. 4C and D). As a result, SINPs + Laser or M@SINPs + Laser treatment significantly increased the M1: M2 macrophage ratio when compared with all other groups (Fig. 4E). We examined the intratumoral ROS production in tumors of mice *i.v.* administrated with SINPs + Laser or M@SINPs + Laser. Figure S9 showed that compared with PBS group, obvious ROS production (green fluorescence) could be observed on the tumor slices collected from the mice of SINPs + Laser or M@SINPs + Laser treatment group, respectively. The above results indicated that under the laser irradiation, SINPs or M@SINPs could generate ROS at tumor sites, which could promote TAMs M2-to-M1 polarization in tumor site. Moreover, the highest M1-to-M2-macrophges ratio was found in the M@SINPs + Laser group (2.27%), which was to be 4.1- and 2.0-fold higher than those of M@SINPs without laser irradiation group (0.56%) and SINPs + Laser group (1.16%), respectively, implying that M@SINPs + Laser treatments significantly promote the re-education of M2-TAMs. Additionally, as illustrated in Fig. 4F and I, the level of M1-associated cytokines including TNF-α and IL-6 in the M@SINPs + Laser group were upregulated by 6.85- and 13.3-fold, respectively, compared to the PBS group, whereas M2-associated cytokines including IL-10 and TGF-b were downregulated. Simultaneously, the most CD8^+^ T cell and CD4^+^ T cell infiltration in tumors were observed in the M@SINPs + Laser group (Fig. 4J and K, S10). Moreover, M@SINPs + Laser treatment increased the CD8^+^ T cells/Tregs ratios by 7.9 times compared with the PBS group, suggesting the reversal of the immune-suppressive microenvironment (Fig. 4L). The intratumoral infiltration of CD206^+^, iNOS^+^, and CD8^+^ cells was further validated by immunofluorescence staining. M@SINPs + Laser group resulted in the lowest CD206 expression and the highest iNOS and CD8 expression, which was consistent with the FCM analysis results in all treatment groups (Fig. 4M). Taken together, these data provide evidence of the remodulation of tumor immune microenvirionment by M@SINPs + Laser treatment, which apparently contributed to the most effective inhibition of tumor growth.

### M@SINPs improved efficacy of aPD-1-block immunotherapy

**Fig. 5:**
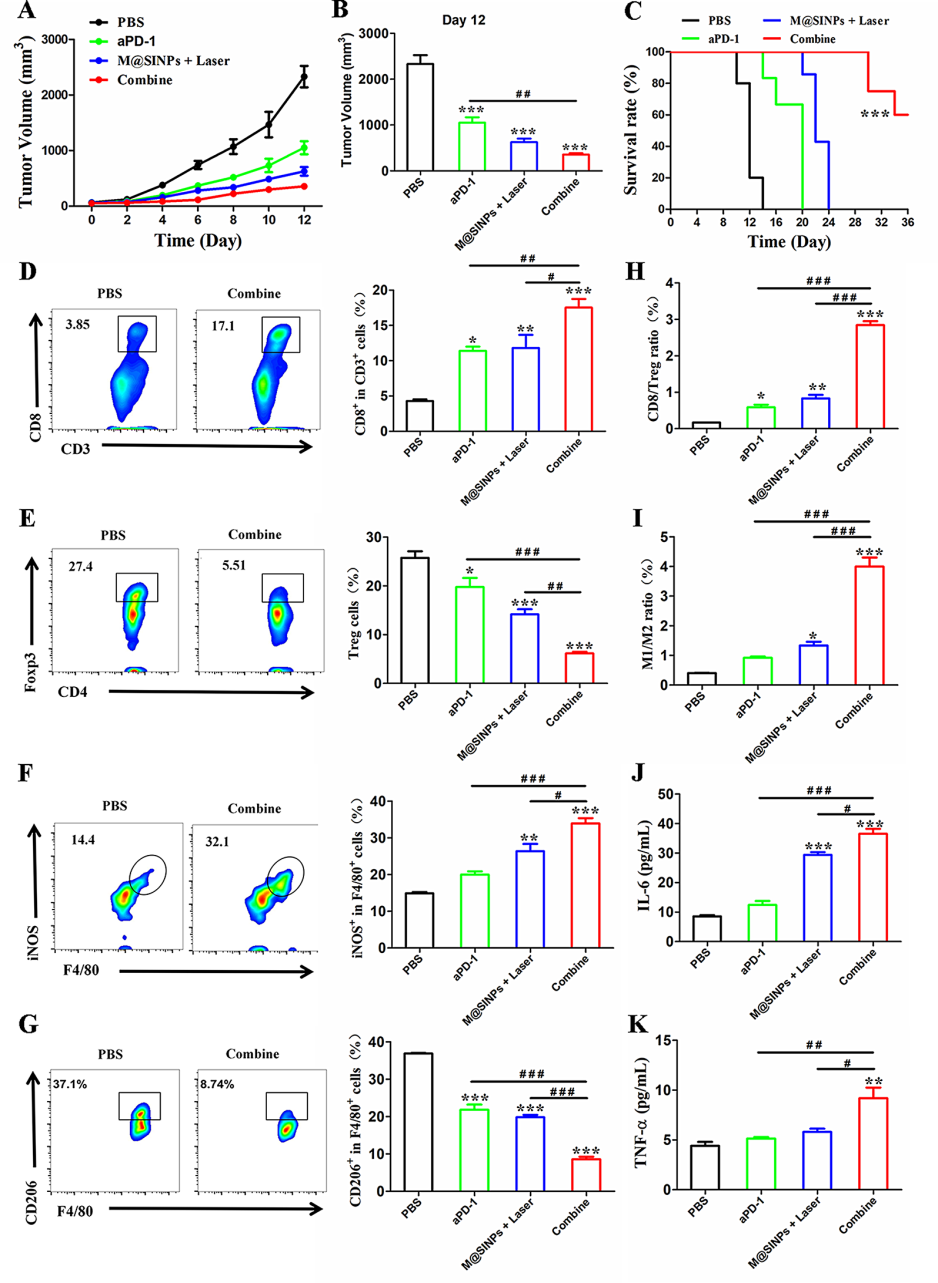
In vivo therapeutic efficacy of M@SINPs in combination with PD-1 blockade. (**A**-**C**) Antitumor effects in terms of tumor growth and survival in CT26-bearing mice with treatment of either PBS, M@SINPs + Laser, aPD-1, or a combination of M@SINPs + Laser and aPD-1 (*n* = 5). (**D**-**G**) Representative FCM plots and quantitative analysis of intratumoral CD8^+^ T cells, Tregs, M1 macrophage (iNOS^+^) and M2 macrophage (CD206^+)^ (*n* = 3). (**H** and **I**) The ratio of CD8/Treg and M1/M2 in the tumor form the treated mice (*n* = 3). (**J** and **K**) Cytokines levels in serum analyzed by ELISA kits (*n* = 3). (#< 0.05, ## < 0.01, ### < 0.001; * vs. PBS, *p < 0.05, **p < 0.01, ***p < 0.001)

TAMs repolarization towards the antitumor M1 phenotype is crucial for improving the therapeutic efficacy of checkpoint inhibitor therapy such as anti-PD-1 antibody (aPD-1) blockade [42, 43]. In this study, we then investigated whether the M@SINPs + Laser treatment in combination with aPD-1 could induce the synergistic antitumor effect. CT26-bearing mice were injected with either PBS, M@SINPs + Laser, aPD-1, or a combination of M@SINPs + Laser and aPD-1. As expected, M@SINPs + Laser treatment showed synergy with aPD-1 in inhibiting tumor growth, and significantly extended the survival of the treated mice (Fig. 5A-C). By analysis of the infiltrating immune cells in tumor tissues after different treatments, we found that the combination-treated tumor had the most intratumoral infiltration of CD8^+^ T cells (17.58%) and M1 macrophages (iNOS^+^, 33.93%), and in turn, the lowest intratumoral infiltration of Tregs (6.16%) and M2-TAMs (CD206^+^, 8.58%) (Fig. 5D-G). As a result, both the CD8/Treg ratio and the M1/M2 ratio in the tumor with the combination treatment increased by more than 4-fold of that in the tumor treated with aPD-1, demonstrating the conversion of “cold tumors” to “hot tumors” (Fig. 5H and I). Additionally, the elevated pro-inflammatory cytokines levels were detected in the combination treatment group, as shown by a 2.9-fold increase in IL-6 and a 1.8-fold increase in TNF-α compared with the aPD-1 group, respectively (Fig. 5J and K). All these data suggested that M@SINPs + Laser treatment improved the antitumor efficacy of the aPD-1 blockade.

## Conclusion

For cancer immunotherapy, TAMs, which play a critical role in the tumor prognosis, are the promising therapeutic target. Here, M@SINPs were designed for TAMs-targeted co-delivery of ROS photogenerator IR820 and SHP2 inhibitor SHP099. These M@SINPs resulted in the concurrent modulation of repolarization and phagocytosis of macrophages and ultimately improved macrophage-based cancer immunotherapy. In vitro assay showed that M@SINPs displayed the higher internalization in macrophages than the nanoparticles without mannose decoration. Upon laser irradiation, M@SINPs can generate the intracellular ROS production and facilitate TAMs repolarization. More importantly, the inhibition of SHP2 could block the CD47-SIRPa pathway and resulted in an enhanced phagocytic potential of macrophages. The in vivo antitumor results indicated that M@SINPs could remodel the tumor’s immune-suppressive microenvironment, including promoting TAMs M2-to-M1 polarization, increasing CTLs infiltration as well as decreasing Tregs in tumor sites, lead to a significant suppression of tumor growth. Furthermore, M@SINPs in combination with aPD-1 could also improve the treatment outcomes of PD-1 blockade and exerted the synergistic antitumor effects. Therefore, M@SINPs hold promise as a strategy to remodel TAMs in TME for improving the antitumor efficiency of conventional and immune checkpoint block therapy.

## Experimental sections

### Nanoparticles preparation and characterization

SNPs were prepared using the desolvation method as described previously [37]. Briefly, 20 mg HSA was dissolved in 0.5 ml of deionized water and adjusted to pH9.0. Then, 0.5 mL of SHP099 in DMSO/H_2_O (1:1, v: v) (2 mg/mL) was added to the above solution and stirred for 10 min. Ethanol was added dropwise until a permanent faint turbidity was obtained. Finally, 20 µL of 8% aqueous glutaraldehyde solution (v/v) was added to harden particles and then under stirring over 24 h. The obtained SNPs were purified by centrifuging, dispersed in deionized water for subsequent experiments. For IR820 loading, SNPs were incubated with 50 µL of IR820-NHS (10 mg/mL) DMSO solution. After stirring for 2 h, SINPs were harvested by centrifuging. Mannose-modified SINPs (M@SINPs) were prepared by mixing SINPs with mannose-NHS for 2 h, then purified and harvested by centrifuging. M@SNPs were obtained by the same preparation procedure of M@SINPs without IR820 loading.

Size and zeta potential of the nanoparticles were determined using a particle size analyzer (Brookhaven 90Plus). The morphology of the nanoparticles was observed with TEM (Tecnai-F20, FEI). Stability assay of the nanoparticles was tested in 10% FBS (pH7.4) at 37 °C. The in vitro release study of IR820 and SHP099 from nanoparticles was carried out in PBS (pH = 7.4) at 37 °C under shaking. The released drug from the nanoparticles at different time intervals was analyzed using UV-*vis*-NIR spectrophotometer for IR820 and HPLC for SHP099, respectively.

### Macrophage uptake in vitro

The internalization of M@SINPs in RAW264.7 cells was evaluated using FCM and CLSM, respectively. M2 polarized (20 ng/mL of IL-4) RAW264.7 cells (in a 12-well plate, 1 × 10^5^ cells/well) were incubated with either free IR820, SINPs or M@SINPs for 4 h (IR820 5.6 ug/mL) and then collected for FCM analysis. Additionally, DAPI staining was performed and the treated cells were observed by CLSM.

### In vitro phagocytosis assay

Mouse colon carcinoma (CT26) cells were labeled with Cell Tracker Green (CTG). 1 × 10^5^ M2 polarized (20 ng/mL of IL-4) RAW264.7 cells were treated with either PBS, free SHP099, SNPs or M@SNPs for 4 h (SHP099 9.9 ug/mL) and then co-cultured with CT26 cells at a ratio of 1:10 in serum-free media for additional 4 h. The cells were collected and stained with fluorescent dye-labeled antibody CD11b^+^, after which the percentage of phagocytosis was measured using a FACSCalibur (BD Biosciences).

For fluorescence microscopic analysis, RAW264.7 macrophages were stained with Rhodaming B and then incubated with different formulations as described above. Following this, CTG-labeled CT26 cells were co-cultured with the treated macrophages for 4 h. Then, CLSM (Zeiss 710) was used to observe the phagocytosis behavior of cancer cells by macrophages.

### Intracellular ROS assay

For ROS observation, RAW264.7 cells were treated with free IR820, SNPs, SINPs and M@SINPs (IR820 5.6 ug/mL) for 4 h in 12-well plates. The cells were rinsed and stained with 2’,7’-dichlorofluorescein diacetate (DCFH-DA). Then, the cells were irradiated with or without 808 nm laser (90 s, 0.5 W/cm^2^), stained with DAPI, and observed by CLSM.

### In vitro macrophage polarization

M2 polarized (20 ng/mL of IL-4) RAW264.7 cells were treated with PBS, free IR820 + free SHP099 (808 nm, 90 s, 0.5 W/cm^2^), SNPs, SINPs, M@SINPs, SINPs + Laser (808 nm, 90 s, 0.5 W/cm^2^) and M@SINPs + Laser (808 nm, 90 s, 0.5 W/cm^2^) at an IR820 concentrationg of 5.6 ug/mL and a SHP099 concentration of 9.9 ug/mL. After incubation for 24 h, the cells were rinsed and labeled with BV421-CD86 and BV650-CD206, and then analyzed using a FACSCalibur (BD Biosciences). Cell culture supernatants in the different treated groups were also collected to determine cytokines levels with ELISA kits.

### CT26-tumor bearing mice

All animal experiments were conducted following Peking Union Medical College & Chinese Academy of Medical Sciences and complied with all relevant ethical norms. Female BALB/c mice (6–8 weeks) were purchased from Huafukang Co., Ltd (Beijing, China). CT26-bearing mice were established by inoculation on the right flank (1 × 10^7^ cells/mouse).

### In vivo biodistribution evaluation

CT26-bearing mice (~ 200 mm^3^) were randomly separated into thee groups and intravenously administered with free IR820, SINPs and M@SINPs (IR820 6.7 mg/kg). The real-time biodistribution was imaged using live animals by PhotonIMAGER optima system at different time points. In addition, tumor tissues were collected at 12 h post-injection for determining the intratumoral drug distribution.

### In vivo therapeutic efficacy study

CT26-bearing mice (~ 50 mm^3^) were used for in vivo therapy and intravenously administrated with PBS, free IR820 + free SHP099 + Laser, SNPs, SINPs, SINPs + Laser, M@SINPs and M@SINPs + Laser at an identical drug dose (IR820 6.7 mg/kg, SHP099 11.8 mg/kg) (every 2 days for 3 doses). For laser group, tumor sites were irradiated with a 808 nm laser (0.5/cm^2^, 90s) at 12 h post-injection. Tumor volumes and body weights were recorded every other day. By the time of sacrificing, major organs were collected for histological analysis.

FCM was used to analyze the immune cells infiltration in the excused tumor samples using a series of fluorophore-labeled antibodies: CTLs (CD45^+^CD3^+^CD8^+^), CD45^+^CD3^+^CD4^+^ T cells, Tregs (CD45^+^CD4^+^Foxp3^+^) T cells, M1-TAMs (CD45^+^F4/80^+^iNOS^+^), M2-TAMs (CD45^+^F4/80^+^CD206^+^). Moreover, the CTLs, M1-TAMs and M2-TAMs infiltration in tumor tissues were examined by immunofluorescence assay. Cytokines in blood was determined using ELISA kits.

### Combinational therapeutic effect with aPD-1

CT26-bearing mice (~ 50 mm^3^) were divided into four groups: PBS, M@SINPs + Laser, aPD-1, and combination (aPD-1 + M@SINPs + Laser). The administration of M@SINPs were the same as above. aPD-1 was dosed by intraperitoneal injection (10 mg/kg, every 3 days for 3 doses). Tumor volumes and body weights were recorded every other day. Besides, the mouse survival rate was recorded. The intratumoral immune cells infiltration including CTLs, Tregs, TAMs were examined by FCM analysis as described above.

### Statistical analysis

Statistical significance of differences was performed by one-way ANOVA followed by Tukey’s post hoc test or the log-rank test using GraphPad Prism 5.0 software. A *p* value of < 0.05 was considered statistically significant.

### Electronic supplementary material

Below is the link to the electronic supplementary material.

Supplementary Material 1

## Data Availability

Data is provided within the manuscript or supplementary information files.
