# Supplementary material for: Engineering nanoparticles-enabled tumor-associated macrophages repolarization and phagocytosis restoration for enhanced cancer immunotherapy
Source: J Nanobiotechnology. 2024 Jun 18;22:341. doi: 10.1186/s12951-024-02622-1 (PMC11184870; doi:10.1186/s12951-024-02622-1)
Supplement: Supplementary file 1 — Supplementary Material 1 [file 12951_2024_2622_MOESM1_ESM.pdf]

Supporting Information

Engineering nanoparticles-enabled tumor-associated macrophages repolarization and phagocytosis restoration for enhanced cancer immunotherapy

Yonghua Gong[#], Wenyue Gao[#], Jinyang Zhang, Xia Dong*, Dunwan Zhu*, Guilei Ma*

Key Laboratory of Biomaterials and Nanotechnology for Cancer Immunotherapy, Tianjin Key Laboratory of Biomaterials, Institute of Biomedical Engineering, Peking Union Medical College & Chinese Academy of Medical Sciences, Tianjin, 300192, China

Yonghua Gong# and Wenyue Gao# contributed equally

[*]***Correspondence to***:

Xia Dong (E-mail: dongxia@bme.pumc.edu.cn)

Dunwan Zhu (E-mail: zhudunwan@bme.pumc.edu.cn)

Guilei Ma (E-mail: magl@bme.pumc.edu.cn)

Table of Contents

1. Experimental sections S3

1.1 Materials and reagents S3

1.2 Experimental section S3

2. Supporting Figures S4

Supplementary Fig. 1 .... S4

Supplementary Fig. 2 .... S4

Supplementary Fig. 3 .... S5

Supplementary Fig. 4 .... S5

Supplementary Fig. 5 .... S6

Supplementary Fig. 6 .... S6

Supplementary Fig. 7 .... S7

Supplementary Fig. 8 .... S7

Supplementary Fig. 9 .... S8

Supplementary Fig. 10 .... S8

Supplementary Fig. 11 .... S9

Supplementary Tab.I S9

1. Experimental sections

1.1 Materials and reagents

Albumin from human serum (Sigma-Aldrich), IR820-HNS and mannose-NHS (Xi'an Ruixi biological technology Co., Ltd), SHP099 (Shanghai Yuanye Bio-Technology Co., Ltd), fluorochrome-labeled CD86, CD206, CD45, CD3, CD4, CD8 and Foxp3, F4/80, iNOS monoclonal antibodies (eBioscience), ELISA kits for TNF-α, TGF−β, IL-10 and IL-6 analysis (eBiosciences). All other chemicals used were of the highest commercially available quality.

BALB/c mice (6-8 weeks old) (Huafukang Laboratory Animal Technology Co., Ltd). All animal experiments were conducted following Peking Union Medical College Guidelines and complied with all relevant ethical norms.

1.2 In vitro cytotoxicity study

To evaluate the photocytotoxicity of the prepared nanoparticles, RAW264.7 cells were treated with either free IR820, SNPs, SINPs or M@SINPs and allowed to incubate for 4 h at 37 °C at at an IR820 concentrationg of 5.6 ug/mL and a SHP099 concentration of 10 ug/mL for 24 h. The cells were replaced with a fresh medium and illuminated at 0.5 W/cm^2 808 nm laser every 30 s for 2 min. Then, the cells were further incubated for 24 h, and a CCK-8 assay kit was used to assay the cell viability.

1.3 In vivo ROS generation in the tumor sites

To detect ROS generation induced by laser irradiation *in vivo*, CT26-tumor bearing mice (~200 mm^3) were received treatments intravenously with PBS, free IR820, SINPs and M@SINPs. The treated mice were then injected intratumorally with dichlorodihydrofluorescein diacetate at 12 h post-injection. Tumors were illuminated at 808 nm laser at 0.5 w/cm^2 for 90s. After that, tumors were separated, frozen sectioned, and observed using CLSM after DAPI staining.

2. Supporting Figures

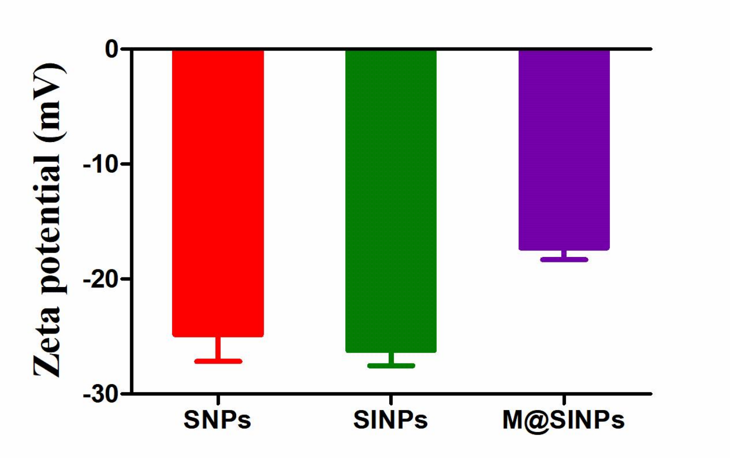

Supplementary Fig. 1. Zeta potentials of the prepared nanoparticles (n = 3).

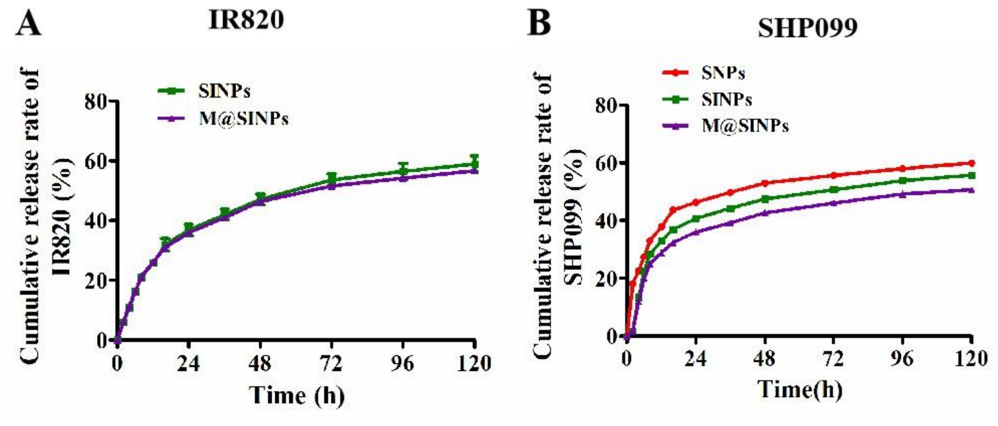

Supplementary Fig. 2. The release profiles of (A)IR820 and (B)SHP099 from the prepared nanoparticles in PBS at pH7.4 (n = 3).

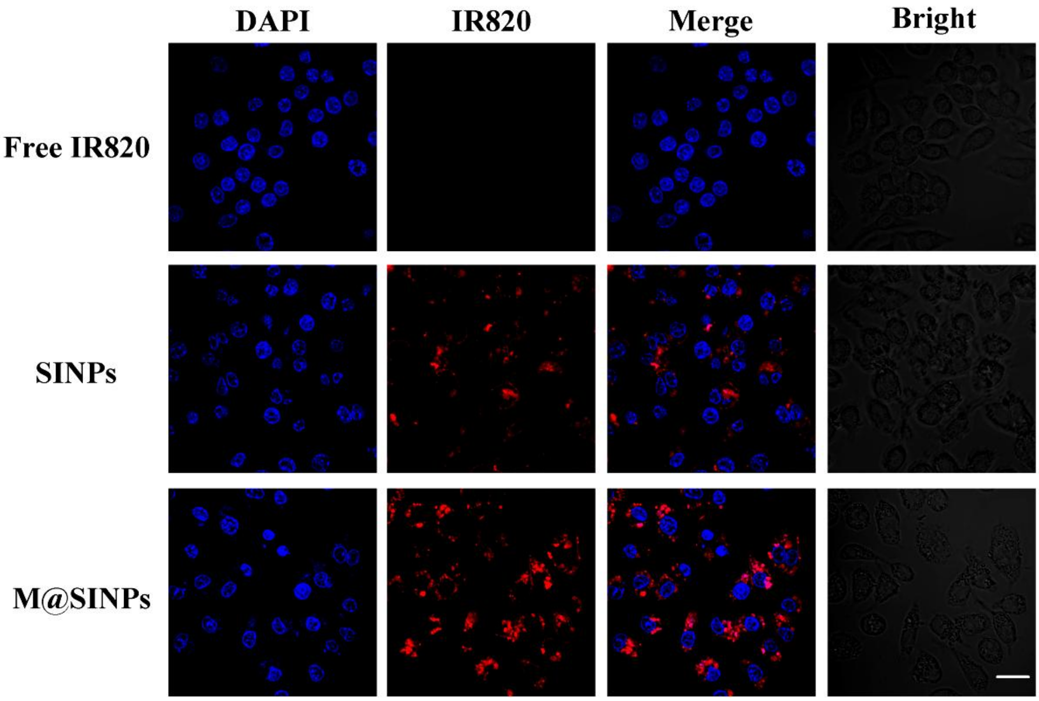

Supplementary Fig. 3. CLSM images showing the internalization of free IR820, SINPs and M@SINPs in RAW264.7 cells (Scale bar, 20 μm).

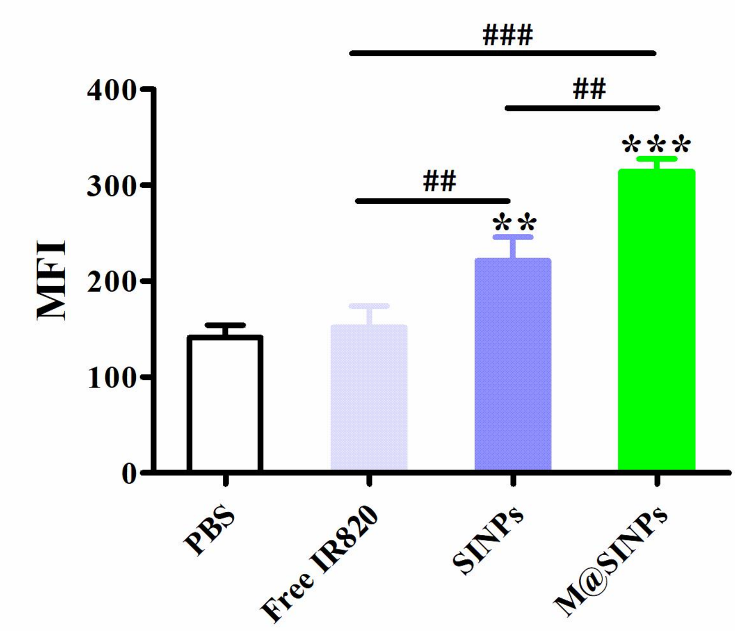

Supplementary Fig. 4. Flow cytometric analysis of the internalization of TAMs in tumor site of the CT26 tumor-bearing mice with *i.v.* injection of free IR820, SINPs and M@SINPs (n = 3)(## < 0.01; * *vs*. PBS, ***p* < 0.01, ****p* < 0.001).

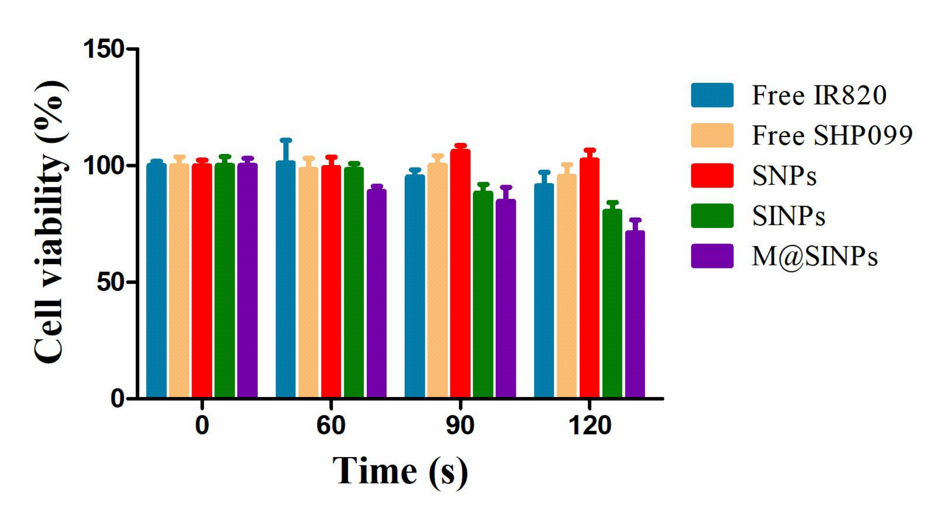

Supplementary Fig. 5. Photocytotoxicity of all the formulations in RAW264.7 cells under laser irradiation (808 nm, 0.5 W/cm^2) (n = 3).

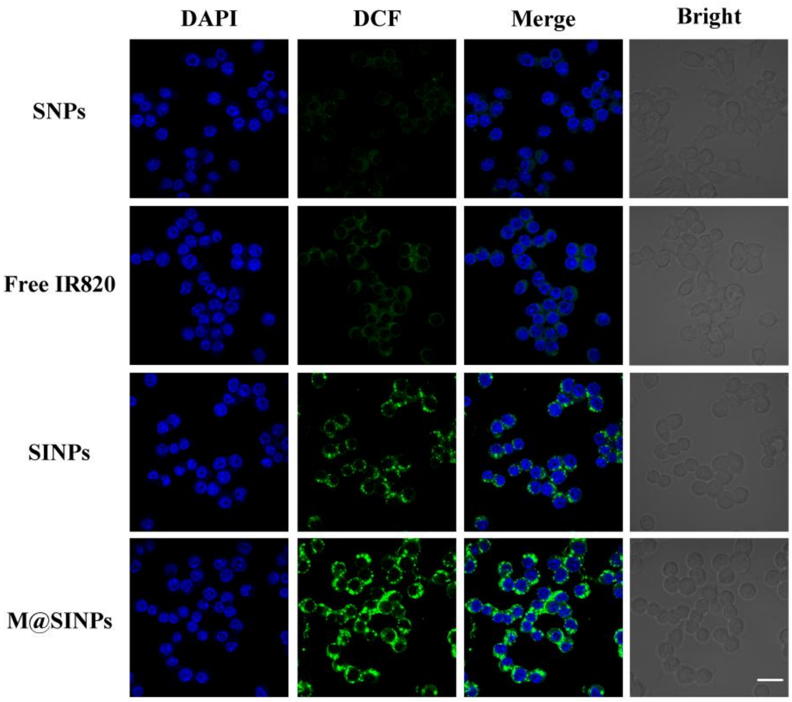

Supplementary Fig. 6. CLSM images of intracellular ROS generation under laser irradiation (808 nm, 0.5 w/cm^2, 90s) in the nanoparticles-treated RAW264.7 cells (Scale bars = 20 μm).

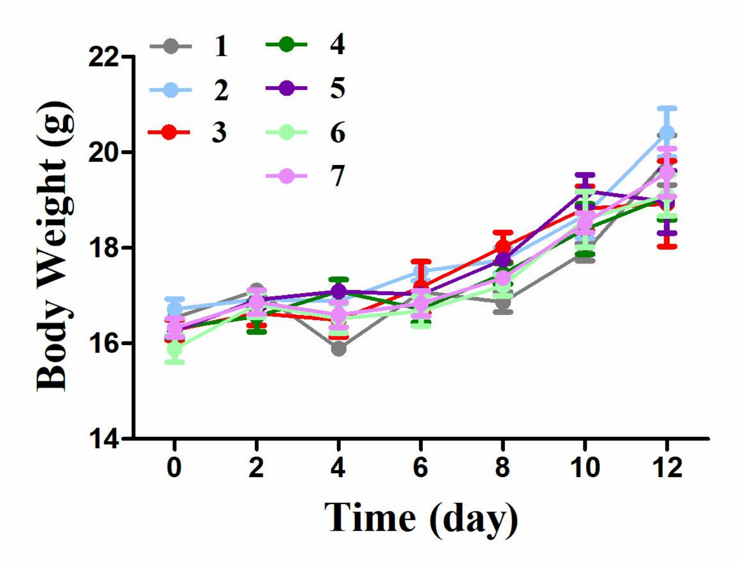

Supplementary Fig. 7. Change of body weight of each group after different treatments indicated (1.PBS, 2.free IR820 + free SHP099 + Laser, 3.SNPs, 4.SINPs, 5.M@SINPs, 6. SINPs + Laser and 7.M@SINPs + Laser) (n = 5).

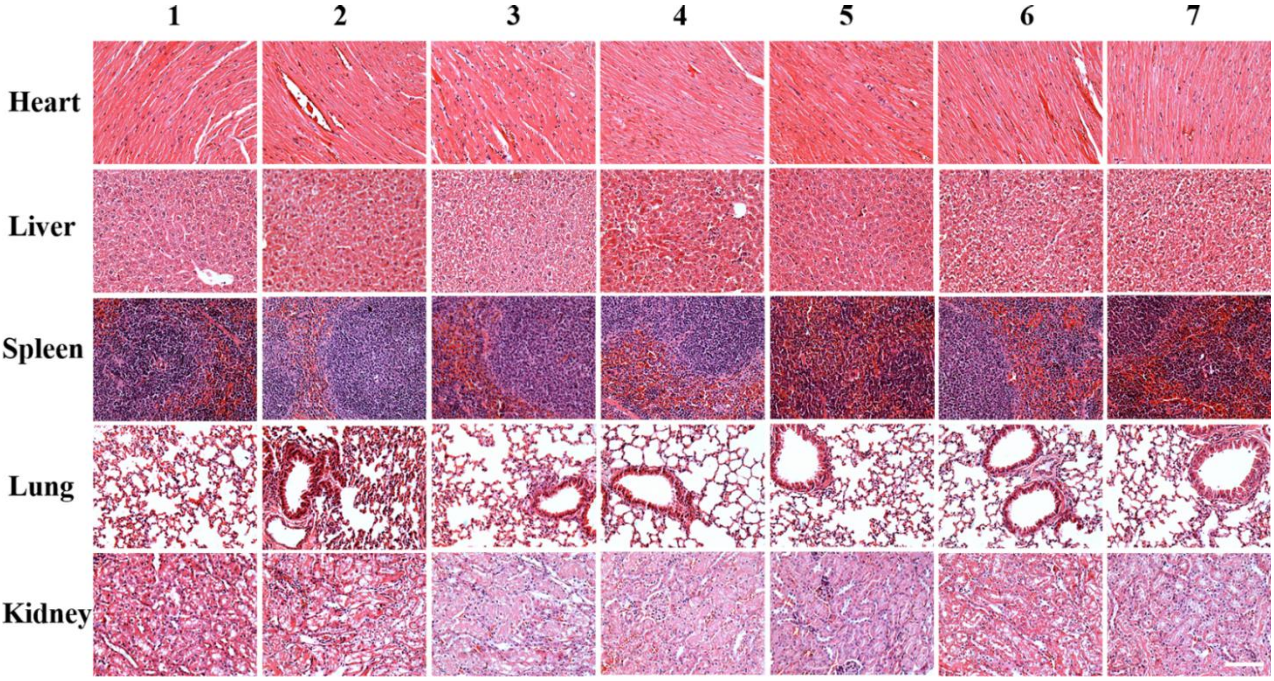

Supplementary Fig. 8. H&E stained images of major organs slices collected from the treated mice (1.PBS, 2.free IR820 + free SHP099 + Laser, 3.SNPs, 4.SINPs, 5.M@SINPs, 6. SINPs + Laser and 7.M@SINPs + Laser) .

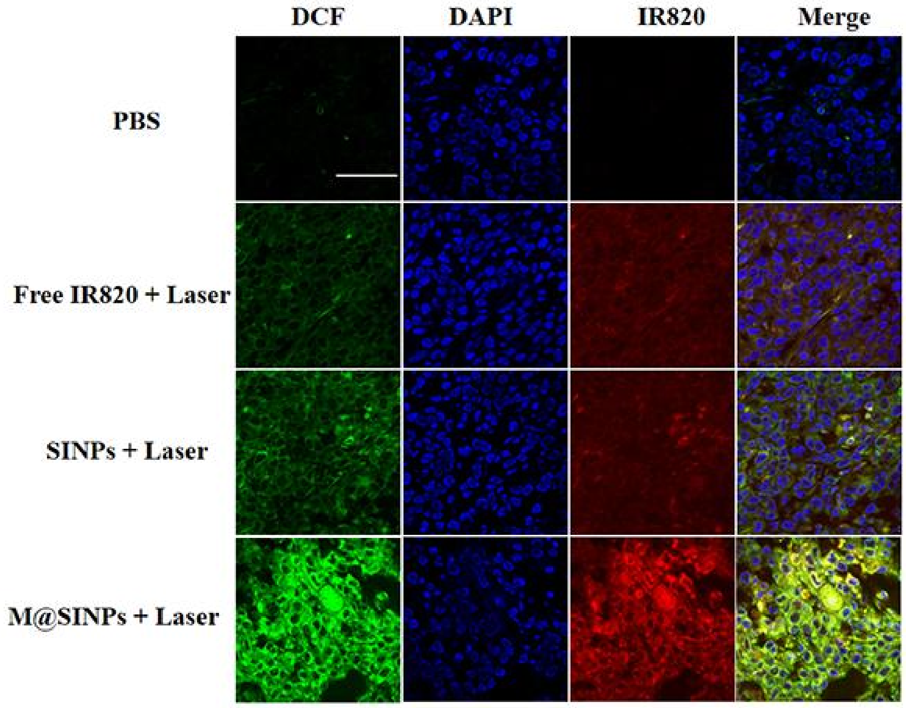

Supplementary Fig. 9. CLSM images showing the intratumoral ROS generation in the CT26-bearing mice receiving the different treatments (laser treatment: 808 nm, 0.5 W/cm^2, 90s) (Scale bars = 20 μm).

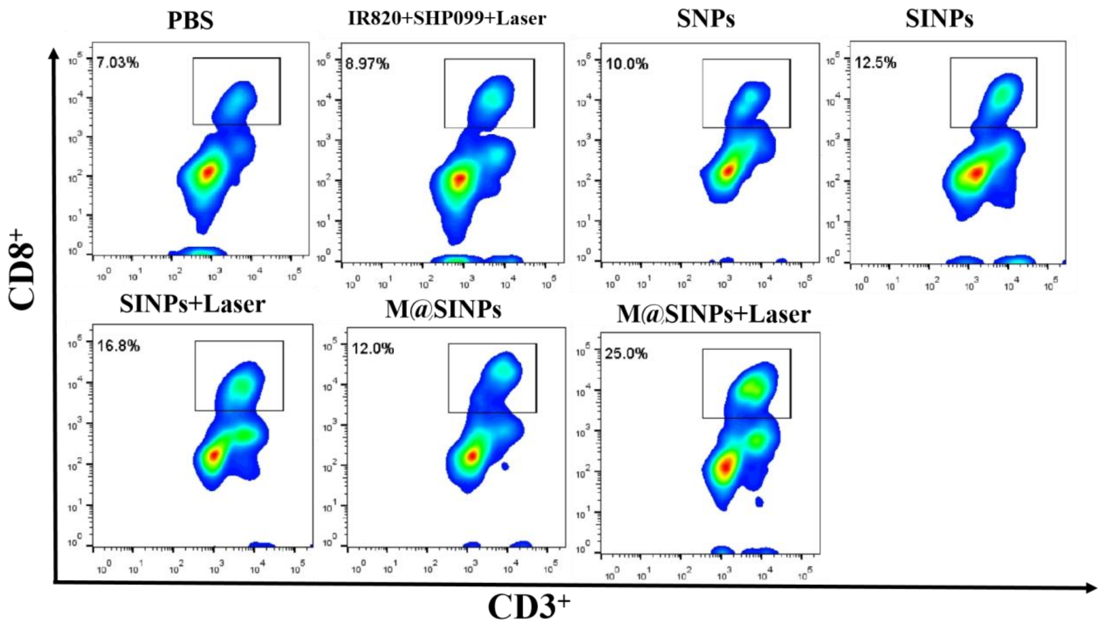

Supplementary Fig. 10. Representative FCM plots and quantitative analysis of intratumoral CD8$^+$ T cells in the CT26-bearing mice receiving the different treatments.

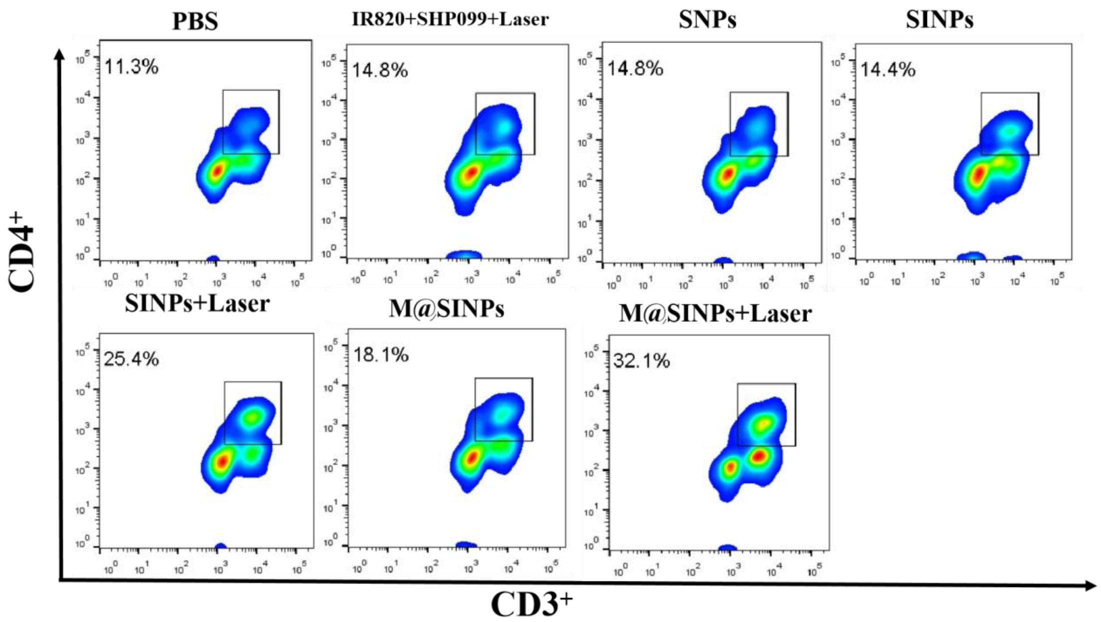

Supplementary Fig. 11. Representative FCM plots and quantitative analysis of intratumoral $CD4^+$ T cells in the CT26-bearing mice receiving the different treatments.

Supplementary Table I. Characterization of the prepared nanoparticles. Values shown are mean ± SD (n = 3).

**Nanoparticle**	**Size (nm)**	**PDI**	**Zeta potential (mV)**	**Drug loading (μg/mg)**	**Entrapment efficiency（%）**
SNPs	142.6 ± 2.7	0.087 ± 0.004	-24.8± 4.0	SHP099:41.9 ± 0.2	SHP099:83.8
SINPs	139.5 ± 3.2	0.081 ± 0.02	-26.2 ± 2.3	IR820:24.7 ± 0.1<br>SHP099:43.5 ± 0.1	IR820:98.9<br>SHP099:87.1
M@SINPs	141.5 ± 2.8	0.105 ± 0.01	-17.3 ± 1.7	IR820:24.7 ± 0.1<br>SHP099:43.2 ± 0.3	IR820:98.9<br>SHP099:86.4
